# Exercise for tension-type headache: protocol for a systematic review and meta-analysis with meta-regression and assessment of exercise reporting completeness

**DOI:** 10.1136/bmjopen-2026-122182

**Published:** 2026-10-20

**Authors:** Ana Bravo-Vazquez, Carlos Bernal-Utrera, Ernesto Anarte-Lazo, Cleofas Rodriguez-Blanco, Deborah Falla

**Affiliations:** 1 Doctoral Program in Health Sciences, University of Seville, Seville, Spain; 2 Faculty of Nursing, Physiotherapy and Podiatry, Physiotherapy Department, University of Seville, Seville, Spain; 3 Fisiosur I+D, Research Institute, Garrucha, Spain; 4 Faculty of Health, UNIE University, Madrid, Spain; 5 Centre of Precision Rehabilitation for Spinal Pain (CPR Spine), School of Sport, Exercise and Rehabilitation Sciences, University of Birmingham, Birmingham, UK

**Keywords:** Exercise, Tension-Type Headache, REHABILITATION MEDICINE

## Abstract

**Abstract:**

**Introduction:**

Tension-type headache (TTH) is the most prevalent primary headache disorder worldwide and represents a significant source of disability. Exercise is increasingly used for the management of TTH due to its potential to induce long-term relief and promote self-management. Although previous systematic reviews suggest beneficial effects of exercise on headache outcomes, substantial heterogeneity limits the interpretation of findings. One possible explanation is the variability and poor reporting of exercise interventions across studies. The Consensus on Exercise Reporting Template (CERT) provides a standardised approach to assess reporting completeness, but its integration with meta-analytic methods remains limited.

**Methods and analysis:**

This protocol describes a systematic review and meta-analysis of randomised controlled trials evaluating the effectiveness of exercise in adults with TTH. Electronic databases (PubMed, Embase, Scopus, Web of Science, PEDro and Cochrane Central Register of Controlled Trials (CENTRAL)) will be searched from inception to June 2026. Two reviewers will independently perform study selection, data extraction and risk of bias assessment using the Cochrane Risk of Bias 2 (RoB 2) tool. The completeness of exercise reporting will be assessed using the CERT checklist. Primary outcomes will include headache frequency and pain intensity; secondary outcomes will include disability, quality of life and medication use. Random-effects meta-analyses will be conducted, with statistical heterogeneity assessed using Cochran’s Q, I² and, where applicable, variance components from multilevel models. Exploratory meta-regression analyses will examine whether reporting completeness (CERT scores), exercise dose and intervention type are associated with treatment effects. The certainty of evidence will be evaluated using the Grading of Recommendations Assessment, Development and Evaluation (GRADE) approach.

**Ethics and dissemination:**

Ethical approval is not required for this study, as it involves a systematic review of published data. The results will be disseminated through publication in a peer-reviewed journal and presentation at scientific conferences.

**PROSPERO registration number:**

CRD420261377102.

**STRENGTHS AND LIMITATIONS OF THIS STUDY**

- The review will include only randomised controlled trials using predefined Population, Intervention, Comparator, Outcomes and Study (PICOS) eligibility criteria.
- Two independent reviewers will conduct study selection, data extraction, risk of bias assessment and exercise reporting completeness assessment, resolving disagreements through consensus or a third reviewer.
- The risk of bias will be assessed using the Cochrane RoB 2 tool, the certainty of evidence using Grading of Recommendations Assessment, Development and Evaluation (GRADE), and the completeness of exercise intervention reporting using the Consensus on Exercise Reporting Template.
- Substantial heterogeneity across exercise interventions, populations, outcome measures and follow-up periods, as well as a potentially low number of eligible studies, may limit the feasibility of meta-analysis and planned subgroup or meta-regression analyses.
- Variability and incomplete reporting of exercise intervention characteristics in the studies may limit the interpretation and reproducibility of the findings.

## Introduction

 Tension-type headache (TTH) is the most prevalent primary headache disorder worldwide and represents a major source of disability, with a substantial impact on quality of life and socioeconomic burden.^1 2^ Despite its high prevalence, the clinical management of TTH remains heterogeneous, partly due to its multifactorial nature and the potential contribution of musculoskeletal, neurophysiological and biopsychosocial factors.^3 4^

Within this context, physiotherapy has emerged as a relevant therapeutic approach,^5 6^ including interventions such as manual therapy, dry needling and exercise.^7^ While several physiotherapy interventions may provide short- to medium-term symptom relief,^8 9^ exercise has attracted increasing interest as a non-pharmacological strategy because of its capacity to address modifiable physical and behavioural factors and potentially promote long-term adaptations.^6 10–12^

Recent systematic reviews and meta-analyses have reported that exercise may reduce headache frequency, pain intensity and disability in individuals with TTH.^10 13^ However, these findings are frequently accompanied by substantial heterogeneity, which limits the interpretability and clinical applicability of pooled estimates.^14^ Understanding the sources of this heterogeneity is essential to improve evidence synthesis and guide clinical decision-making.

One potential explanation for this variability is the inconsistency and insufficient reporting of exercise interventions across studies. Key aspects such as type, intensity, progression and supervision are often poorly described, making it difficult to determine whether interventions are comparable. This lack of transparency may mask important clinical differences between studies, contribute to unexplained heterogeneity and limit reproducibility and implementation in clinical practice.^15–17^

To address these issues, the Consensus on Exercise Reporting Template (CERT) was developed as a standardised tool to evaluate the completeness of exercise intervention reporting. Differences in reporting completeness may also provide information about the extent to which relevant intervention characteristics can be identified and examined across trials, although reporting completeness should not be interpreted as a proxy for intervention efficacy or methodological quality.^15^ Although CERT has been increasingly applied in rehabilitation research, its integration with meta-analytic approaches remains limited.^18–20^

To our knowledge, no systematic review has simultaneously evaluated the effectiveness of exercise for TTH, assessed the completeness of reporting using CERT and explored the potential influence of reporting completeness on treatment effects and between-study heterogeneity through meta-regression analyses.

## Objectives

The aim of this systematic review is to evaluate the effectiveness of exercise for adults with TTH and to explore potential sources of heterogeneity across studies.

The specific objectives are:

- To evaluate the effectiveness of exercise on headache frequency and pain intensity in patients with TTH.
- To assess the completeness of reporting of exercise interventions using CERT.
- To explore whether completeness of exercise intervention reporting, assessed using CERT scores, is associated with treatment effect estimates and between-study heterogeneity in outcomes such as headache frequency, pain intensity and disability through exploratory meta-regression analyses.

## Methods and analysis

### Study design and registration

This protocol has been developed in accordance with the Preferred Reporting Items for Systematic Review and Meta-Analysis Protocols (PRISMA-P) guidelines.^21^ The systematic review will be conducted following the Preferred Reporting Items for Systematic Reviews and Meta-Analyses (PRISMA) 2020 statement.^22^ The protocol has been registered in the International Prospective Register of Systematic Reviews (PROSPERO) (CRD420261377102). Any amendments to the protocol will be documented and updated in the PROSPERO record.

### Eligibility criteria

Eligibility criteria will be defined according to the Population, Intervention, Comparator, Outcomes and Study design framework.^23^

#### Population

Studies including adults (≥18 years) diagnosed with episodic or chronic TTH based on the International Classification of Headache Disorders criteria (third edition or earlier validated versions) will be eligible.^24^ Studies including mixed headache populations will be included only if data for TTH can be extracted separately.

#### Intervention

Studies evaluating exercise as a primary intervention will be included. Exercise is defined as structured, planned and repetitive physical activity aimed at improving physical function and health outcomes.^16^ This includes, but is not limited to, strengthening exercises, motor control training, aerobic exercise and multimodal exercise programmes. Studies comparing different types of exercise interventions (eg, strengthening vs motor control, aerobic vs resistance or multimodal approaches) will also be included. Studies combining exercise with other interventions will be included only if the exercise component is clearly described and represents a central element of the intervention. Exercise interventions will be pre-specified and classified into categories (eg, motor control, strengthening, aerobic, multimodal) to enable planned subgroup and meta-regression analyses.

#### Comparator

Eligible comparators will include no intervention, usual care, placebo or sham interventions and other active treatments, including alternative exercise interventions. Comparator type will be recorded and considered as a potential source of clinical heterogeneity. Where a sufficient number of independent randomised controlled trials (RCTs) is available, subgroup analyses will be conducted according to comparator type. If the number of independent RCTs is insufficient to support reliable comparator-specific analyses, eligible comparator groups may be pooled for the primary meta-analysis when considered clinically and methodologically appropriate, and comparator-specific findings will be interpreted descriptively.

#### Outcomes

Primary outcomes will include headache frequency and pain intensity. Secondary outcomes will include headache-related disability, quality of life and medication consumption. Studies must report at least one of the primary outcomes to be included.

#### Study design

Only RCTs published in peer-reviewed journals will be included. No restrictions will be applied regarding publication date, geographic location or healthcare setting. Studies published in English or Spanish will be included.

### Exclusion criteria

Studies involving patients with other primary or secondary headache disorders (eg, migraine) will be excluded unless data for TTH are reported separately.

RCTs for which the full text is not available, including those published solely in abstract form, will be excluded.

### Information sources and search strategy

A comprehensive systematic search will be conducted in the following electronic databases: PubMed (MEDLINE), Embase, Scopus, Web of Science (Science Citation Index Expanded), PEDro and the Cochrane Central Register of Controlled Trials (CENTRAL), from inception to June 2026.

The search strategy will combine controlled vocabulary (eg, Medical Subject Headings (MeSH) terms) and free-text terms related to TTH and therapeutic exercise. Boolean operators (AND/OR) will be used to combine search terms. The full search strategy for each database can be found in online supplemental material.

In addition, grey literature will be explored, including clinical trial registries, reference lists of included studies and citation tracking of relevant articles. Experts in the field may be contacted to identify additional studies if necessary.

### Study selection and data extraction

All identified records will be imported into Zotero and duplicates will be removed prior to screening. The deduplicated records will then be exported to Rayyan for the screening process. Study selection will be conducted in two stages: first, titles and abstracts will be screened, followed by full-text assessment of potentially eligible studies.

Two independent reviewers (AB-V and EA-L) will perform the screening process. Any disagreements will be resolved through discussion and consensus, and if necessary, by consulting a third reviewer (DF). The study selection process will be reported using a PRISMA flow diagram.

Data will be extracted independently by the same two reviewers (ABV and EA-L) using a standardised data extraction form. Extracted information will include study characteristics (author, year, country), participant characteristics (sample size, age, diagnostic criteria), intervention details (type, frequency, intensity, duration), comparator details and outcome data.

When necessary, study authors will be contacted to obtain missing or unclear information.

### Dose

Total prescribed exercise exposure will be calculated as prescribed exercise frequency (sessions/week) × session duration (minutes/session) × intervention duration (weeks), yielding total prescribed exercise minutes. Where frequency, session duration or intervention duration are reported using different units, these will be converted to common units before calculation. This variable will represent prescribed exercise exposure in minutes and will not be interpreted as a comprehensive measure of physiological training load. Where sufficient independent RCTs and variability are available, total prescribed exercise exposure will be explored as a continuous study-level moderator in exploratory meta-regression.

### Harmonised exercise intensity score

Exercise intensity will be extracted according to the method reported in each study, including percentage of one-repetition maximum (%1RM), rating of perceived exertion (RPE), Borg scales, prescribed load or qualitative descriptors. Where exercise intensity is reported using heterogeneous methods across studies, a prespecified Harmonised Exercise Intensity Score (HEIS) will be used as an exploratory harmonisation tool.

The HEIS will classify exercise intensity into three ordinal categories: 1=low intensity, 2=moderate intensity and 3=high intensity. Where %1RM is reported, intensity will be classified as low (<40%), moderate (40–60%) or high (>60%). For the Borg 6–20 scale, values<11 will be classified as low, 11–13 as moderate and ≥14 as high. For the Borg CR10 scale, values 1–3 will be classified as low, 4–6 as moderate and 7–10 as high. Where studies use explicit qualitative descriptors (low, moderate or high intensity), these descriptors will be retained according to the terminology reported by the study.

The method used to report exercise intensity will also be coded separately as intensity type: 1=RPE-based, 2=%1RM or load-based, 3=qualitative descriptor and 4=intensity not reported. The original intensity measure and descriptor reported by each study will be retained in the extraction database to allow verification of the harmonisation process.

The HEIS has not been previously validated and will therefore be interpreted solely as an exploratory harmonisation tool rather than a validated measure of exercise intensity. It will only be used in exploratory subgroup or meta-regression analyses when sufficient information and variability are available.

### Exercise frequency and duration

Exercise frequency and session duration will be extracted in their original units and converted to sessions per week and minutes per session, respectively, where necessary. Intervention duration will be converted to weeks. These variables will be used to calculate total prescribed exercise dose as described above. Where sufficient information and variability are available, total exercise dose will be examined as a continuous study-level moderator.

### Multimodal interventions

For interventions comprising multiple exercise components, the principal exercise component will be identified according to the predefined exercise categories. Where one component clearly represents the predominant therapeutic exercise component, the intervention will be assigned to that category. When no single component can be considered predominant, the intervention will be classified as multimodal. The individual components and their reported frequency, duration and intensity will nevertheless be extracted where available. Total exercise dose will be calculated based on the overall prescribed exercise programme when the necessary information is available.

### Risk of bias assessment

The risk of bias in included RCTs will be assessed independently by two reviewers using the Cochrane Risk of Bias 2 (RoB 2) tool.^25^ Any disagreements will be resolved through discussion and consensus, and if necessary, by consulting the third reviewer.

The RoB 2 tool evaluates bias across the following domains: bias arising from the randomisation process, bias due to deviations from intended interventions, bias due to missing outcome data, bias in measurement of the outcome and bias in selection of the reported result. Each domain will be rated as ‘low risk of bias’, ‘some concerns’ or ‘high risk of bias’, and an overall risk of bias judgement will be provided for each study.

The risk of bias assessment will be incorporated into the interpretation of the results and considered in sensitivity analyses. The certainty of evidence will be assessed using the Grading of Recommendations Assessment, Development and Evaluation (GRADE) approach,^26^ with all evaluations performed independently by two reviewers and disagreements resolved by consensus or consultation with a third reviewer. Cohen’s kappa statistic index will be calculated to report reliability.^27^

### Exercise reporting completeness assessment

The completeness of reporting of exercise interventions will be assessed using the CERT. The CERT is a standardised tool designed to evaluate the completeness and transparency of exercise intervention reporting.^15^ Two independent reviewers will apply the CERT checklist to each included study. Any discrepancies will be resolved through discussion and consensus, and if necessary, by consulting the third reviewer.

The CERT consists of 16 items covering key domains of exercise prescription, including type of exercise, dosage, progression, supervision and adherence. Each item will be scored as ‘yes’ (clearly reported), ‘no’ (not reported) or ‘not applicable’, and a total score will be calculated for each study.

Complete reporting of exercise interventions may facilitate the identification and interpretation of potentially relevant intervention characteristics, such as exercise type, frequency, intensity, duration, progression, supervision and adherence. Consequently, completeness of reporting may be associated with variation in observed treatment effects because studies with more complete reporting may provide greater information about intervention characteristics that could contribute to differences in effect estimates. However, reporting completeness is not equivalent to intervention quality or treatment efficacy, and any association between CERT scores and effect estimates may be explained by other study-level characteristics.

CERT scores will be used to describe the completeness of exercise intervention reporting and, where sufficient independent RCTs are available, will be explored as an exploratory moderator of treatment effects in meta-regression analyses. CERT scores reflect the completeness and transparency of reporting rather than the efficacy or methodological quality of the exercise intervention itself. Therefore, an association between CERT scores and treatment effects, if observed, will not be interpreted as evidence that better reporting results in greater treatment efficacy. Any observed association may reflect differences in intervention characteristics that are more completely reported, but may also be influenced by study-level characteristics such as publication year, journal reporting standards, research group characteristics, sample size or methodological quality. CERT-based meta-regression will therefore be considered hypothesis-generating and interpreted cautiously, with particular attention to the potential for confounding and ecological bias.

### Inter-rater reliability

Inter-rater agreement will be assessed for study selection, risk-of-bias assessment, CERT assessment and GRADE certainty assessment. Cohen’s kappa statistic will be calculated to quantify agreement between reviewers. For ordinal assessments, including ordinal GRADE judgments where appropriate, weighted Cohen’s kappa will be used. 95% CIs will be calculated when estimable.

### Outcomes

The primary outcomes of this systematic review will be headache frequency and pain intensity in adults with TTH. Headache frequency will be defined as the number of headache days or episodes over a specified time period (eg, per week or per month), as reported in the included studies. Pain intensity will be measured using validated scales such as the Visual Analogue Scale, Numerical Rating Scale or equivalent instruments reported by the original studies.

When multiple assessment time points are reported, outcomes will be analysed separately according to predefined assessment periods: post-intervention, short-term follow-up, medium-term follow-up and long-term follow-up. Post-intervention will be defined as the assessment performed immediately after completion of the intervention and within the first week. Short-term follow-up will be defined as an assessment performed at or beyond 7 days after the post-intervention assessment and at or before 3 months after the intervention. Medium-term follow-up will be defined as an assessment performed more than 3 months and before 6 months after the intervention. Long-term follow-up will be defined as an assessment performed at or beyond 6 months after the intervention. Where the same outcome is reported at multiple eligible time points, each assessment will be assigned to the corresponding predefined temporal category and analysed separately rather than combining assessments across different follow-up periods.

Secondary outcomes will include any headache-related outcomes assessed using validated instruments. We will be open to all outcome domains measured with validated tools, including (but not limited to) headache-related disability, health-related quality of life and medication consumption. Disability may be assessed using instruments such as the Headache Impact Test-6 or the Migraine Disability Assessment, as well as any other validated measures used in TTH populations. Health-related quality of life will be considered when evaluated using validated generic or condition-specific instruments.

Effect sizes will be calculated using mean differences or standardised mean differences (SMD) with 95% CIs, depending on the measurement scales used across studies. When multiple scales are used, standardised mean differences will be prioritised for pooling.

### Data synthesis and statistical analysis

A narrative synthesis will initially be undertaken to summarise the characteristics of the included studies, participant populations, exercise interventions, comparator conditions and outcome measures. Quantitative synthesis will be considered when at least two clinically comparable studies report the same outcome at the same assessment time point. Clinical comparability will be assessed considering the study population, intervention characteristics, comparator condition and outcome construct before pooling.

Continuous outcomes will be synthesised using SMDs, expressed as Hedges’ g with 95% CIs, to allow pooling when the same underlying construct is assessed using different instruments or scales. Where appropriate, outcomes measured using the same instrument and scale may be synthesised using mean differences. Outcome measures will be directionally harmonised so that negative effect estimates consistently favour exercise.

When studies report multiple eligible comparisons, all relevant comparisons will be retained where clinically appropriate. Statistical dependence between effect estimates originating from the same study will be accounted for in the meta-analysis rather than treating each comparison as an independent study. When a study contributes more than one eligible comparison to a meta-analysis, a three-level random-effects model will be considered, with effect estimates nested within studies. This approach will allow both between-study and within-study variability to be considered while retaining relevant comparisons. Where appropriate, intervention groups may be combined when this is necessary to avoid double-counting participants and when the interventions are sufficiently similar to justify combination.

Random-effects meta-analyses will be conducted using R (R Foundation for Statistical Computing), with the meta package or other appropriate statistical packages as required. Restricted maximum likelihood will be used to estimate variance components where applicable, and 95% CIs for pooled effects will be calculated using the Hartung-Knapp adjustment. For analyses involving multiple comparisons from the same study, clustered or three-level random-effects models will be used where appropriate to account for the dependence between effect estimates.

Outcomes will be harmonised before pooling when different labels represent the same underlying construct. For example, measures of headache intensity assessed using different validated instruments, such as the Visual Analogue Scale or Numerical Rating Scale, will be considered measures of pain intensity when they assess the same underlying construct. Similarly, headache frequency measures reported using different terminology will be considered for pooling when they represent the same underlying construct. Measures representing distinct constructs will be retained separately. Instruments will not be reclassified solely on the basis of their name; their intended construct and the definition provided by the original study will be considered when determining outcome equivalence.

Separate meta-analyses will be performed according to the predefined temporal categories: post-intervention, short-term follow-up, medium-term follow-up and long-term follow-up. Where the same outcome is reported at several eligible time points within or across these periods, assessments will be analysed according to their corresponding predefined temporal category rather than combining different follow-up periods.

Statistical heterogeneity will be evaluated using Cochran’s Q statistic and the I² statistic. For clustered or three-level models, the estimated variance components (τ²) will also be examined to distinguish between-study and within-study variability where estimable. Heterogeneity will be interpreted according to recommendations from the Cochrane Handbook for Systematic Reviews of Interventions, while considering the magnitude and precision of the pooled effect estimates and the clinical characteristics of the included studies. The interpretation of I² will not be based solely on fixed numerical thresholds.^28^

Exploratory random-effects meta-regression will be conducted when a sufficient number of independent RCTs is available for the specific outcome and assessment period, and when sufficient variability in the moderator is present. Meta-regression will only be undertaken when at least five independent RCTs contribute to the relevant analysis. This threshold will be considered a minimum feasibility criterion for exploratory analysis rather than an indication of adequate statistical power. The number of independent RCTs, rather than the number of effect estimates or comparisons, will be considered when determining whether meta-regression is feasible.

The CERT total score will be examined as a continuous study-level moderator. Exercise type will also be considered as a categorical moderator according to the predefined exercise categories. Interventions will be assigned to the exercise category that best represents the principal therapeutic exercise component. Multicomponent interventions will be classified according to their predominant exercise component when this can be clearly established; otherwise, they will be retained as multimodal interventions. Additional exploratory meta-regression analyses will examine total prescribed exercise dose and exercise intensity where sufficient information and variability are available. Categorical moderators will not be examined when the distribution of studies across categories is too sparse to support a meaningful exploratory comparison.

Meta-regression analyses will be conducted as univariable models, and multivariable meta-regression will not be undertaken. All meta-regression analyses will be considered exploratory and hypothesis-generating because of the limited number of contributing studies, potential limitations in statistical power and the possibility of ecological bias and confounding by other study-level characteristics. Associations identified through meta-regression will therefore not be interpreted as individual-level or causal relationships. The potential influence of individual studies, the distribution and variability of the moderators, and the number of contributing independent RCTs will also be considered when interpreting the findings. Results based on the minimum number of eligible RCTs will be interpreted cautiously.

Sensitivity analyses will be conducted to assess the robustness of the findings to important methodological assumptions. Where sufficient studies are available, meta-analyses will be repeated after excluding studies judged to be at high risk of bias. The corresponding random-effects model will then be refitted using the remaining studies.

Influence analyses will also be considered using a leave-one-study-out approach, in which each study is removed in turn and the corresponding meta-analysis is refitted. Where studies contribute multiple comparisons, all comparisons originating from the same study will be removed together to preserve the dependence structure of the data. These analyses will be used to assess whether pooled effect estimates or estimates of heterogeneity are substantially influenced by individual studies.

Potential small-study effects will be explored visually using funnel plots when at least 10 independent RCTs are available for a given meta-analysis. Egger’s test will also be considered when at least 10 independent RCTs are available and the assumptions required for its application are met. When fewer than 10 independent RCTs are available, funnel plots and Egger’s test will not be performed because their interpretation and statistical power are likely to be limited. Any evidence of funnel-plot asymmetry will be interpreted cautiously, as asymmetry may arise from factors other than publication bias.

All data will be managed using standardised extraction forms in Microsoft Excel or equivalent software. Statistical analyses will be performed using R. All analytical decisions, deviations from the prespecified methods, and data-handling procedures will be documented to ensure transparency and reproducibility.^28^

### Patients and public involvement

Patients and members of the public were not directly involved in the conduct, data analysis or reporting of this systematic review. However, the research question was informed by discussions held within patient and public involvement meetings at the Centre of Precision Rehabilitation for Spinal Pain, University of Birmingham. These discussions contributed to the identification and prioritisation of the research topic but did not constitute direct patient or public involvement in the conduct of the review. Findings will be shared with these groups and through broader dissemination activities.

### Ethics and dissemination

This study is based on the analysis of previously published data and does not involve direct participation of human subjects. Therefore, ethical approval is not required.

The findings of this systematic review will be disseminated through publication in a peer-reviewed scientific journal and presentation at relevant national and international conferences. In addition, results may be shared with clinical and academic audiences involved in headache research and physiotherapy practice, as well as with the patient and public involvement groups that contributed to the identification and prioritisation of the research topic.

## Discussion

This systematic review and meta-analysis aims to evaluate the effectiveness of exercise for adults with TTH,^1 2^ while also exploring potential sources of heterogeneity across studies. A key strength of this review is the integration of exercise reporting completeness, assessed using the CERT,^15–17^ with quantitative synthesis methods. This approach may provide novel insights into whether variability in intervention reporting contributes to differences in treatment effects.^9 10 13^

By incorporating meta-regression analyses, this study will explore the extent to which factors such as reporting completeness, exercise dose and intervention type influence outcomes. This may help to better understand inconsistencies observed in previous reviews and support more precise interpretation of evidence.

Some limitations are anticipated, including potential heterogeneity in interventions and outcome measures, as well as variability in reporting completeness across studies. Nonetheless, the findings of this review may contribute to improving both the design and reporting of exercise interventions in future research, as well as informing clinical practice.

## Supplementary material

10.1136/bmjopen-2026-122182 online supplemental file 1
