# Supplementary material for: Exercise for tension-type headache: protocol for a systematic review and meta-analysis with meta-regression and assessment of exercise reporting completeness
Source: BMJ Open. 2026 Oct 20;16(10):e122182. doi: 10.1136/bmjopen-2026-122182 (PMC13684226; doi:10.1136/bmjopen-2026-122182)
Supplement: Supplementary file 1 [file bmjopen-2026-122182.reviewer_comments.pdf]

PEER REVIEW HISTORY

BMJ Open publishes all reviews undertaken for accepted manuscripts. Reviewers are asked to complete a checklist review form and are provided with free text boxes to elaborate on their assessment. These free text comments are reproduced below.

ARTICLE DETAILS

Title (Provisional)

Exercise for tension-type headache: Protocol for a systematic review and meta-analysis with meta-regression and assessment of exercise reporting completeness

Authors

Bravo-Vazquez, Ana; Bernal-Utrera, Carlos; Anarte-Lazo, Ernesto; Rodriguez-Blanco, Cleofas; Falla, Deborah

---

VERSION 1 - REVIEW

**Reviewer**	**1**
**Name**	**Gjestvang, Christina**
**Affiliation**	**Norwegian School of Sports Sciences, Department of Sports Medicine**
**Date**	**09-Sep-2026**
**COI**	**None**

Thank you for letting me review this protocol that addresses an important and clinically relevant topic. The protocol is well structured and follows established methodological standards for systematic reviews. However, several aspects of the proposed methodology require further clarification and justification before the protocol can be considered sufficiently robust. Below are my comments divided into major and minor comments. Addressing my issues would strengthen the protocol and credibility of the eventual review findings.

Major comments

1. Page 6, lines 29-34: The eligible comparators represent fundamentally different contrasts and are likely to produce different effect sizes. The authors should therefore clarify whether all comparator groups will be pooled together, whether subgroup analyses will be conducted according to comparator type, and whether comparisons against alternative exercise interventions will be analyzed separately from comparisons against non-exercise controls. Without such clarification, substantial clinical heterogeneity may be introduced into the pooled analyses.

2. Page 8, lines 53-58: The authors intend to use the total CERT score as an explanatory variable in meta-regression. CERT was developed as a reporting quality tool, not as a measure of intervention quality. Thus, better reporting does not necessarily reflect a more effective intervention. Higher CERT scores may be associated with publication year, journal standards, or research group characteristics rather than true treatment effects. The authors should discuss these conceptual limitations more explicitly and justify why CERT score is expected to function as a plausible moderator of treatment effect.

3. Page 9, lines 12-13: The authors state that post-intervention and closest follow-up data will be prioritized, but it remains unclear which time point will be considered as the primary analysis, how studies reporting multiple follow-up assessments will be managed, and whether short-, medium-, and long-term outcomes will be analyzed separately. These decisions should be specified a priori.

4. Page 10, lines 15-30: The authors plan to perform meta-regression using CERT score, exercise dose, and intervention type as moderators. However, it is not specified the minimum number of studies required per covariate. To my knowledge, this research field consists of a small number of RCTs. If only a limited number of studies are eligible, meta-regression results may be unstable and potentially misleading. I recommend that the authors clarify the minimum number of studies required per covariate (e.g., ≥10 studies), whether univariable or multivariable meta-regression models will be used, how multiple analyses will be managed, and how ecological bias will be addressed beyond the brief statement currently provided.

5. Page 10, lines 17-20: The protocol states that exercise frequency, intensity, and duration will be analyzed as dose-related variables, but it does not explain how these variables will be standardized across studies, how multimodal interventions will be handled, and whether a measure of total training load or exercise volume will be calculated. Given the considerable variation in exercise reporting across trials, a more detailed analytical plan is needed.

6. Page 10, lines 29-30: The authors propose using funnel plots and Egger's test but do not specify a minimum number of studies required for these analyses. I recommend aligning the protocol with current methodological guidance and explicitly stating that such analyses will only be performed when a sufficient number of studies are available.

Minor comments

7. Page 7, lines 12-17 and Supplementary Material S1: The main text lists PubMed, Scopus, Web of Science, PEDro, and CENTRAL. However, the supplementary material additionally presents an Embase search strategy. It is therefore unclear whether Embase will be formally searched and included in the review. This should be clarified.

8. Page 8, lines 28-29: The authors state that Cohen's kappa statistics will be calculated. However, they do not specify which review processes will be evaluated (e.g., study

screening, risk of bias assessment, CERT scoring, or all of these). Additional details would improve transparency.

9. Page 10, lines 44-51 and Page 11, lines 3-4: The authors state that the research question originated from discussions within patient and public involvement meetings at the University of Birmingham. However, you later state that no patient or public involvement was included in the design, conduct, reporting, or dissemination plans of the study. These statements appear contradictory and should be reconciled or explained more clearly.

10. Reference list: A substantial proportion of the rationale is supported by publications authored by members of the study team. The manuscript would be strengthened by drawing more extensively on independent sources to support the background and rationale for the review.

11. Supplementary Material S1: The search strategy includes few exercise-related terms. The authors should consider incorporating additional synonyms. You should also indicate whether the search strategy was developed or peer-reviewed by an information specialist or librarian.

VERSION 1 - AUTHOR RESPONSE

Response to Reviewer 1 Comments

We sincerely thank you for your careful and constructive evaluation of our manuscript. We greatly appreciate the thoughtful comments and suggestions, which have helped us to improve the clarity, transparency, and methodological rigor of the protocol. We have carefully considered all comments and have revised the manuscript and supplementary material accordingly. All corresponding changes have been highlighted in yellow for ease of reference, and our responses are summarized below:

Major comments

1.Page 6, lines 29-34: The eligible comparators represent fundamentally different contrasts and are likely to produce different effect sizes. The authors should therefore clarify whether all comparator groups will be pooled together, whether subgroup analyses will be conducted according to comparator type, and whether comparisons against alternative exercise interventions will be analyzed separately from comparisons against non-exercise controls. Without such clarification, substantial clinical heterogeneity may be introduced into the pooled analyses.

Response 1. Thank you for this comment. We agree that the eligible comparator conditions represent clinically distinct contrasts and should not be assumed to be equivalent.

We have therefore clarified the protocol to explicitly distinguish comparisons with non-exercise controls (e.g., no intervention, usual care, placebo or sham interventions) from comparisons with alternative exercise interventions and other active treatments. Comparator type will be recorded and considered as a potential source of clinical heterogeneity. We have also clarified that comparator-specific subgroup analyses will be conducted where a sufficient number of independent RCTs is available to provide meaningful and reasonably precise estimates. We

have prespecified that, when comparator-specific analyses are not feasible, comparator groups may be pooled in the primary meta-analysis where clinically and methodologically appropriate. In such cases, comparator type will be considered when interpreting clinical heterogeneity and the pooled estimates.

These clarifications have been added to the Comparator subsection and the Data synthesis and statistical analysis section of the revised protocol.

2. Page 8, lines 53-58: The authors intend to use the total CERT score as an explanatory variable in meta-regression. CERT was developed as a reporting quality tool, not as a measure of intervention quality. Thus, better reporting does not necessarily reflect a more effective intervention. Higher CERT scores may be associated with publication year, journal standards, or research group characteristics rather than true treatment effects. The authors should discuss these conceptual limitations more explicitly and justify why CERT score is expected to function as a plausible moderator of treatment effect.

Response 2. Thank you for this important conceptual clarification. We agree that the CERT assesses the completeness and transparency of exercise intervention reporting and should not be interpreted as a measure of intervention efficacy, intervention quality, or methodological quality.

We have revised the manuscript accordingly and now consistently refer to CERT scores as a measure of completeness of exercise intervention reporting rather than intervention quality. Our rationale for exploring CERT score as a moderator is not that better reporting is expected to cause greater treatment effects. Rather, more complete reporting may provide greater information about potentially relevant intervention characteristics, such as exercise type, frequency, intensity, duration, progression, supervision, and adherence, which may help explain variation in observed treatment effects across trials. We have also explicitly acknowledged that any observed association between CERT score and treatment effects may be explained by other study-level characteristics, including publication year, journal reporting standards, research group characteristics, sample size, and methodological quality. Therefore, CERT-based meta-regression will be considered exploratory and hypothesis-generating, and any observed association will not be interpreted as evidence of a causal relationship between reporting completeness and treatment efficacy.

These conceptual limitations and the rationale for examining CERT score as an exploratory moderator have now been clarified in the Exercise reporting completeness assessment, Objectives, and Data synthesis and statistical analysis sections of the revised protocol.

3. Page 9, lines 12-13: The authors state that post-intervention and closest follow-up data will be prioritized, but it remains unclear which time point will be considered as the primary analysis, how studies reporting multiple follow-up assessments will be managed, and whether short-, medium-, and long-term outcomes will be analyzed separately. These decisions should be specified a priori.

Response 3. Thank you for this important comment. We agree that the original wording did not sufficiently specify how multiple assessment time points would be handled.

We have therefore clarified the temporal analysis strategy in the revised manuscript. Rather than designating a single follow-up time point as the sole primary temporal analysis, outcomes are analysed separately according to predefined assessment periods: post-intervention, short-term, medium-term, and long-term follow-up. Post-intervention is defined as the assessment performed immediately after completion of the intervention and within the first week; short-term follow-up as more than 7 days after the post-intervention assessment and up to 3 months after the intervention; medium-term follow-up as more than 3 months and less than 6 months; and long-term follow-up as 6 months or longer. Where the same outcome is reported at multiple eligible time points, each assessment is assigned to the corresponding predefined temporal category and analysed separately rather than combining assessments across different follow-up periods.

The corresponding text has been added to the Outcomes and Data synthesis and statistical analysis sections of the revised manuscript.

4. Page 10, lines 15-30: The authors plan to perform meta-regression using CERT score, exercise dose, and intervention type as moderators. However, it is not specified the minimum number of studies required per covariate. To my knowledge, this research field consists of a small number of RCTs. If only a limited number of studies are eligible, meta-regression results may be unstable and potentially misleading. I recommend that the authors clarify the minimum number of studies required per covariate (e.g., ≥10 studies), whether univariable or multivariable meta-regression models will be used, how multiple analyses will be managed, and how ecological bias will be addressed beyond the brief statement currently provided.

Response 4. Thank you for this important methodological comment. We agree that the conditions under which meta-regression is performed should be clearly prespecified, particularly given the relatively small number of RCTs expected in this field.

We have therefore clarified that meta-regression will only be conducted when at least 5 independent RCTs contribute to the relevant outcome and assessment period, and when sufficient variability in the moderator is available. We have chosen 5 independent RCTs as a minimum threshold for exploratory analyses because the number of eligible RCTs in this clinical field is expected to be limited. Importantly, this threshold is considered a minimum feasibility criterion rather than an indication of adequate statistical power. Analyses based on relatively few RCTs will therefore be interpreted as exploratory and hypothesis-generating, with particular caution regarding imprecision, influential studies, ecological bias, and confounding by other study-level characteristics. The number of independent RCTs, rather than the number of effect estimates or comparisons, will be used to determine whether a meta-regression is feasible. Meta-regression analyses will be conducted as univariable models. CERT score will be examined as a continuous moderator, while exercise type, total exercise dose, and exercise intensity will only be examined when sufficient information and variability are available. Multivariable meta-regression will not be undertaken. We have also clarified that categorical moderators will not be examined when their distribution across categories is too sparse to provide a meaningful comparison. The influence of individual studies and the distribution and variability of the moderators will be considered when interpreting the findings, and no association will be interpreted as an individual-level or causal relationship.

These clarifications have been incorporated into the Data synthesis and statistical analysis section of the revised protocol.

5. Page 10, lines 17-20: The protocol states that exercise frequency, intensity, and duration will be analyzed as dose-related variables, but it does not explain how these variables will be standardized across studies, how multimodal interventions will be handled, and whether a measure of total training load or exercise volume will be calculated. Given the considerable variation in exercise reporting across trials, a more detailed analytical plan is needed.

Response 5. We can see now that the protocol required greater detail regarding the standardization and analysis of exercise characteristics across studies.

We have therefore clarified how exercise frequency, session duration, and intervention duration will be standardized and used to calculate total prescribed exercise dose, defined as frequency (sessions/week) × session duration (minutes/session) × intervention duration (weeks). Exercise intensity will be extracted according to the method reported in each study and, where necessary, harmonized using the prespecified Harmonized Exercise Intensity Score (HEIS), while retaining the original intensity descriptor for verification. The HEIS will be used only as an exploratory proxy and will not be interpreted as a validated measure of exercise intensity. We have also clarified how multimodal interventions will be classified for exercise-category analyses. Interventions will be assigned to the predominant exercise component when this can be clearly established; otherwise, they will be classified as multimodal. The individual exercise components will nevertheless be extracted where sufficient information is available, and total prescribed exercise dose will be calculated from the overall programme when the necessary data are reported.

These clarifications have been incorporated into the Dose, Exercise intensity, Exercise frequency and duration, Multimodal interventions, and Data synthesis and statistical analysis sections of the revised protocol.

6. Page 10, lines 29-30: The authors propose using funnel plots and Egger's test but do not specify a minimum number of studies required for these analyses. I recommend aligning the protocol with current methodological guidance and explicitly stating that such analyses will only be performed when a sufficient number of studies are available.

Response 6. We agree that the conditions for assessing small-study effects should be prespecified.

We have therefore clarified that funnel plots and Egger's test will only be considered when at least 10 independent RCTs are available for a given meta-analysis. When fewer than 10 independent RCTs are available, these analyses will not be performed because their interpretation and statistical power are likely to be limited. We have also clarified that any evidence of funnel-plot asymmetry will be interpreted cautiously, as asymmetry may arise from factors other than publication bias.

These clarifications have been incorporated into the Data synthesis and statistical analysis section of the revised protocol.

Minor comments

7. Page 7, lines 12-17 and Supplementary Material S1: The main text lists PubMed, Scopus, Web of Science, PEDro, and CENTRAL. However, the supplementary material additionally presents an Embase search strategy. It is therefore unclear whether Embase will be formally searched and included in the review. This should be clarified.

Response 7. Thank you for identifying this accidental omission. We confirm that Embase was included as one of the electronic databases searched for this review. The main text has been revised accordingly to include Embase among the six databases searched, consistent with the search strategy provided in Supplementary Material S1.

This clarification has been incorporated into the Information sources and search strategy section of the revised protocol.

8. Page 8, lines 28-29: The authors state that Cohen's kappa statistics will be calculated. However, they do not specify which review processes will be evaluated (e.g., study screening, risk of bias assessment, CERT scoring, or all of these). Additional details would improve transparency.

Response 8. We agree that the review processes for which inter-rater agreement will be assessed should be explicitly specified. We have therefore clarified that Cohen's kappa will be used to assess agreement during study selection, risk-of-bias assessment, CERT assessment, and GRADE certainty assessment. Weighted Cohen's kappa will be used for ordinal assessments where appropriate.

These clarifications have been incorporated into a dedicated Inter-rater reliability subsection of the revised protocol.

9. Page 10, lines 44-51 and Page 11, lines 3-4: The authors state that the research question originated from discussions within patient and public involvement meetings at the University of Birmingham. However, you later state that no patient or public involvement was included in the design, conduct, reporting, or dissemination plans of the study. These statements appear contradictory and should be reconciled or explained more clearly.

Response 9. Thank you for identifying this apparent inconsistency. We agree that the previous wording did not clearly distinguish between the discussions that informed the research question and direct patient and public involvement in the conduct of the review.

We have revised the Patient and Public Involvement and Ethics and dissemination section to clarify that the research question was informed by discussions within patient and public involvement meetings, but that patients and members of the public were not directly involved in the conduct, data analysis, or reporting of the review. We have also clarified that findings will be shared with these groups as part of the dissemination process.

These clarifications have been incorporated into the revised protocol.

10. Reference list: A substantial proportion of the rationale is supported by publications authored by members of the study team. The manuscript would be strengthened by drawing more extensively on independent sources to support the background and rationale for the review.

Response 10. We have broadened the reference base and incorporated independent publications where appropriate, particularly for the epidemiological background, clinical characteristics, physiotherapy management, exercise effectiveness, and methodological rationale. Publications authored by members of the study team have been retained where they provide directly relevant evidence to the specific rationale of the present protocol.

11. Supplementary Material S1: The search strategy includes few exercise-related terms. The authors should consider incorporating additional synonyms. You should also indicate whether the search strategy was developed or peer-reviewed by an information specialist or librarian.

Response 11. Thank you for this helpful comment. We have revised the search strategies to broaden the exercise-related terminology and improve the sensitivity of the database searches. Additional synonyms and related terms were incorporated, including terms referring to therapeutic exercise, resistance and strength training, aerobic and endurance exercise, motor control exercise, physical activity, and rehabilitation. We have also clarified the development and review process of the search strategy in Supplementary Material S1. The search strategies were developed by the review team based on the review question, eligibility criteria, and terminology identified from relevant literature, and were reviewed within the review team for consistency, clarity, and completeness. No external information specialist or librarian was involved in the development or peer review of the search strategies.

These changes have been incorporated into the revised Supplementary Material S1.
