# Supplementary material for: Exercise for tension-type headache: protocol for a systematic review and meta-analysis with meta-regression and assessment of exercise reporting completeness
Source: BMJ Open. 2026 Oct 20;16(10):e122182. doi: 10.1136/bmjopen-2026-122182 (PMC13684226; doi:10.1136/bmjopen-2026-122182)
Supplement: Supplementary file 2 [file bmjopen-16-10-s001.docx]

**S1. SEARCH STRATEGY**

**PubMed**

("Tension-Type Headache"[Mesh] OR "tension type headache"[tiab] OR "tension-type headache"[tiab] OR TTH[tiab])

AND

("Exercise Therapy"[Mesh] OR "Motor Activity"[Mesh] OR "Resistance Training"[Mesh] OR "Aerobic Exercise"[Mesh]

OR exercise[tiab] OR exercises[tiab] OR "therapeutic exercise"[tiab] OR "exercise therapy"[tiab]

OR "physical activity"[tiab] OR "physical training"[tiab] OR training[tiab]

OR "resistance exercise"[tiab] OR "resistance training"[tiab] OR "strength training"[tiab]

OR strengthening[tiab] OR "strength exercise"[tiab]

OR "aerobic exercise"[tiab] OR "aerobic training"[tiab]

OR "endurance exercise"[tiab] OR "endurance training"[tiab]

OR "motor control"[tiab] OR "motor control exercise"[tiab]

OR rehabilitation[tiab])

AND

(randomized controlled trial[pt] OR controlled clinical trial[pt] OR random*[tiab] OR RCT[tiab])

AND

("Humans"[Mesh])

AND

("Adult"[Mesh])

**Embase**

('tension type headache'/exp OR 'tension type headache':ti,ab OR 'tension-type headache':ti,ab OR TTH:ti,ab)

AND

('exercise therapy'/exp OR 'physical activity'/exp OR 'resistance training'/exp OR 'aerobic exercise'/exp

OR exercise:ti,ab OR exercises:ti,ab OR 'therapeutic exercise':ti,ab OR 'exercise therapy':ti,ab

OR 'physical training':ti,ab OR training:ti,ab

OR 'resistance exercise':ti,ab OR 'resistance training':ti,ab

OR 'strength training':ti,ab OR strengthening:ti,ab OR 'strength exercise':ti,ab

OR 'aerobic exercise':ti,ab OR 'aerobic training':ti,ab

OR 'endurance exercise':ti,ab OR 'endurance training':ti,ab

OR 'motor control':ti,ab OR 'motor control exercise':ti,ab

OR rehabilitation:ti,ab)

AND

('randomized controlled trial'/exp OR random*:ti,ab OR RCT:ti,ab)

AND

[humans]/lim AND [adult]/lim

**Scopus**

TITLE-ABS-KEY ("tension-type headache" OR "tension type headache" OR TTH)

AND

TITLE-ABS-KEY (

exercise OR exercises OR "therapeutic exercise" OR "exercise therapy"

OR "physical activity" OR "physical training" OR training

OR "resistance exercise" OR "resistance training"

OR "strength training" OR strengthening OR "strength exercise"

OR "aerobic exercise" OR "aerobic training"

OR "endurance exercise" OR "endurance training"

OR "motor control" OR "motor control exercise"

OR rehabilitation

)

AND

TITLE-ABS-KEY (random* OR "randomized controlled trial" OR RCT)

**Web of Science**

TS=("tension-type headache" OR "tension type headache" OR TTH)

AND

TS=(

exercise OR exercises OR "therapeutic exercise" OR "exercise therapy"

OR "physical activity" OR "physical training" OR training

OR "resistance exercise" OR "resistance training"

OR "strength training" OR strengthening OR "strength exercise"

OR "aerobic exercise" OR "aerobic training"

OR "endurance exercise" OR "endurance training"

OR "motor control" OR "motor control exercise"

OR rehabilitation

)

AND

TS=(random* OR "randomized controlled trial" OR RCT)

**Cochrane**

("tension-type headache" OR "tension type headache" OR TTH)

AND

(

exercise OR exercises OR "therapeutic exercise" OR "exercise therapy"

OR "physical activity" OR "physical training" OR training

OR "resistance exercise" OR "resistance training"

OR "strength training" OR strengthening OR "strength exercise"

OR "aerobic exercise" OR "aerobic training"

OR "endurance exercise" OR "endurance training"

OR "motor control" OR "motor control exercise"

OR rehabilitation

)

AND

(randomized OR randomised OR trial OR "controlled trial")

**General study filters**

The following limits will be applied across all databases:

- Population: Human studies only

- Participants: Adults (≥18 years)

- Study design: Randomized controlled trials (RCTs)

- Language: English and Spanish

- Time period: From database inception to June 2026

**Additional notes**

No restrictions will be applied regarding geographical location or healthcare setting.

The search strategy is designed to be highly sensitive to ensure comprehensive identification of randomized controlled trials evaluating therapeutic exercise interventions in tension-type headache.

Grey literature and additional sources (e.g., reference lists, citation tracking, and clinical trial registries) will be used to complement database searches where appropriate.

**Search strategy development and peer review**

The search strategies were developed by the review team based on the review question, the eligibility criteria, and relevant terminology identified from previous literature. The strategies were independently reviewed within the review team for clarity, consistency, and completeness before being finalised. No external information specialist or librarian was involved in the development or peer review of the search strategies.
